# Symptom experiences and influencing factors in patients undergoing chemotherapy for gastrointestinal cancers: a qualitative study

**DOI:** 10.3389/fpsyg.2024.1264275

**Published:** 2024-05-09

**Authors:** Xiaoying Huang, Heng Yang, Yanyan Qiao

**Affiliations:** ^1^Department of Oncology, Longhua Hospital, Shanghai University of Traditional Chinese Medicine, Shanghai, China; ^2^Department of Interventional Radiology, Longhua Hospital, Shanghai University of Traditional Chinese Medicine, Shanghai, China

**Keywords:** chemotherapy, gastrointestinal cancer, symptom experiences, influencing factor, qualitative interview

## Abstract

**Objective:**

To explore the symptom experiences and influencing factors of gastrointestinal (GI) cancer patients on chemotherapy (CTX) in China.

**Methods:**

Semi-structured interviews were conducted with 13 GI cancer patients undergoing CTX. Following the Colaizzi 7-step analysis method, the interview data were read carefully, meaningful statements related to the research questions were extracted, coded, collected, and described in detail, and the authenticity of the theme was verified.

**Results:**

Nine themes were grouped into two main areas including the characteristics of symptom experiences and influences on symptom experiences.

**Conclusion:**

The symptom experiences of patients undergoing CTX for GI cancer is poor and influenced by multiple factors. Nurses need to pay attention to the assessment and monitoring of CTX-related symptoms, improve symptom recognition, enhance doctor-patient communication and social support, explore intelligent management methods, and increase the efficiency of healthcare services to improve patients’ symptom experience.

## 1 Introduction

Global incidence cases, death cases, and disability-adjusted life-years (DALYs) of gastrointestinal (GI) cancers showed an overall ascending trend over the past 30 years (Xie et al., 2022). It was estimated that more than 1.9 million new cases of colorectal cancer (including anus) and 935,000 deaths occurred in 2020. Colorectal cancer ranks third in incidence but second in mortality. There are more than 1 million new cases of stomach cancer and 769,000 deaths, ranking fifth in incidence and fourth in mortality (Sung et al., 2021). The IARC predicts that the burden will increase by more than 60% by 2030 (Arnold et al., 2017).

Chemotherapy (CTX) is one of the primary treatment modalities for GI cancers (Moertel, 1978). CTX helps to reduce mortality and prolong survival in patients with GI cancers (Bokemeyer and Honecker, 2003). However, CTX has some side effects, which can be immediate, short-term, or long-term (Fox et al., 2017; Akbarali et al., 2022). Several quantitative studies have shown that patients with GI cancer undergoing CTX experience a range of symptoms associated with toxicity and side effects, such as pain, nausea, numbness/tingling, fatigue, sleep disturbances, etc. (Pettersson et al., 2014; Tantoy et al., 2018). Despite professional guidance and management of patients with CTX, symptoms are often poorly controlled (Devlin et al., 2017; Fox et al., 2017). Inadequate treatment of symptoms has a significant impact on several fronts: it results in patients suffering from related changes in physical health, mental status, health behaviors, personal identity, and economic status, prevents patients from receiving effective treatments, and increases mortality rates (Tan et al., 2022; Farrell et al., 2023; Wilson et al., 2023).

In recent years, scholars have conducted numerous quantitative studies on the symptoms of GI cancer patients undergoing chemotherapy (Pettersson et al., 2014; Tantoy et al., 2018). However, there is a dearth of qualitative study on patients’ symptom experiences. Therefore, we conducted interviews with patients undergoing CTX for GI cancer. We wanted to gain a deeper understanding of the symptoms and distress experienced by patients during CTX, and identify the factors that influence their symptom experiences. This will help healthcare professionals to provide more comprehensive management and support to patients, thereby enhancing treatment outcomes and quality of life for patients.

## 2 Methods

### 2.1 Study design

This study used semi-structured interviews to identify patients’ experiences of symptoms and the factors that influence them. This design was chosen to comprehend the situation of CTX-related symptoms in patients with GI cancer from their personal experiences, which would help the researchers to obtain data on patients’ experiences that cannot be captured through quantitative studies.

### 2.2 Participants

The study sample selected GI cancer patients hospitalized for CTX between September and December 2022. Purposive sampling was used based on the characteristics of patients, such as the gender, age, educational level, cancer site, and disease course of cancer patients. We selected the study subjects based on the principle of maximum differentiation. The eligibility criteria were as follows: patients diagnosed with GI cancer; received CTX or adjuvant CTX; could read and communicate in Chinese (Mandarin); not currently receiving hospice care; had a prognosis of 6 months or longer; and showed no evidence of dementia or delirium, as determined by a medical chart or clinical consensus of physicians.

### 2.3 Data collection

We used the descriptive phenomenological method of qualitative research. A semi-structured interview outline (Table 1) was formulated according to the research purpose, including literature reading, expert discussion, and pre-interview of 2 patients. One-to-one, face-to-face interviews were conducted by one experienced research specialist nurse in a sunroom, and the environment was quiet and not disturbed. Before the interview, she explained the research purpose, methods, and the importance of transcripts and recordings, promised to protect their privacy, and asked for the interview for 0.5 ~ 1 h. After the interview, the reflection diary should be written in time, and the text materials should be returned to the interviewees for confirmation.

**Table 1:** Examples of interview questions.

Questions
What symptoms did you experience during chemotherapy and how did you feel?
What symptoms worried you or were difficult to control?
How have your symptoms affected your life or work?
What do you think influenced your symptoms?

### 2.4 Data analysis method

Data analysis was conducted by HXY and QYY using Colaizzi’s 7-step methodology (Elo and Kyngäs, 2008): (1) transcribing the audio of the interviews over a 24-h period using NVivo 8. 0 software; (2) close reading of the transcribed text to gain a deeper understanding of the patient’s experience; (3) analysis of the transcripts sentence by sentence and coding for key messages; (4) team discussion and integration of initial themes; (5) further analysis to accurately reflect the patient’s experience; (6) aggregation of themes and sub-themes to construct a complete understanding; and (7) a return visit to the patient by telephone to verify the accuracy of the analysis. Patients confirmed the analyses and provided new feedback, pointing out new factors that had an impact on symptom experience. We incorporated this new information into the study to assess its specific role for patients.

### 2.5 Ethical considerations

This study complied with the requirements of the Declaration of Helsinki. The study was approved by the Ethics Committee of Longhua Hospital, affiliated with the Shanghai University of Traditional Chinese Medicine. The approval number was 2022LCSY079. The purpose of the study was briefly explained to respondents when they were recruited. Written informed consent forms were distributed and collected from these patients upon their agreement to participate in the interview. The permission to audio-record the interview was also obtained from the patients before the interview.

## 3 Results

The sample size was set by information saturation (Guest et al., 2006). Eventually, 13 patients were included in this study. The general information about the respondents is shown in Table 2.

**Table 2:** Patients’ characteristics.

ID	Gender	Age	Educational level	Marital status	Medical insurance condition	Diagnosis	Cancer stage	Frequency of CTX
N1	Female	48	Master	Married	Have insurance	COAD	IV	36
N2	Female	74	Uneducated	Married	Have insurance	READ	III	30
N3	Male	69	Primary school	Married	Have insurance	COAD	IV	11
N4	Female	72	Uneducated	Married	Have insurance	STAD	III	7
N5	Male	60	High school	Married	No insurance	COAD	IV	21
N6	Male	65	Junior college	Married	Have insurance	READ	IV	19
N7	Female	69	Primary school	Married	Have insurance	STAD	IV	15
N8	Male	66	Middle school	Married	Have insurance	COAD	IV	21
N9	Male	70	University	Married	Have insurance	COAD	IV	3
N10	Male	60	High school	Married	Have insurance	STAD	IV	10
N11	Male	70	Primary school	Married	No insurance	READ	IV	1
N12	Female	19	High school	Single	No insurance	STAD	IV	8
N13	Male	29	University	Single	No insurance	COAD	IV	9

COAD, Colon cancer; READ, Rectum cancer; STAD, Gastric cancer.

The interview data were collated and analysed to categorise 9 themes in 2 main areas, characteristics of symptom experiences and influencing factors of symptom experiences.

### 3.1 Characteristics of symptom experiences

This section outlines 3 first level themes and 10 s level themes to provide a more comprehensive understanding of the patient’s symptom experience. Details are shown in Table 3.

**Table 3:** Characteristics of symptom experiences.

First-level theme	Second-level theme	Patient quote
Diversity of symptom types	Coexistence of multiple physical symptoms	N11: “I experienced a range of physical symptoms including nausea, fatigue, and loss of appetite. It felt like my body was constantly fighting something.”
	Persistent health concerns leading to psychological burden	N8: “I’m nervous, ouch… As long as there is any physical discomfort, I will rush to see a doctor, especially afraid of whether the tumor is metastatic recurrence.”
	Interrelated symptoms forming complex symptom clusters	N1: “I have a stomachache at night, nausea and vomiting, and I cannot sleep. I always seem in a daze when I get up the next day.”
Dynamics of symptom experiences	Individual differences in symptom intensity	P6: “The guy I had chemo with, same drugs, he barely reacted, but I felt very tired.”
	Inconsistent onset and duration of symptoms	N3: “I had a little bit of a poor appetite after CTX. Yesterday I ate pretty well. Today is the second day. I have not had lunch yet. My nausea was much better when I got home. Just a lack of energy.”
	Symptoms change cyclically	P8: “My fatigue and nausea symptoms tend to come in waves. Symptoms get worse after receiving treatment and then ease up when I go home”
Interference with daily life	Symptoms affect self-care ability	N4: “Doing housework is tough, and I lack the energy.”
	Symptoms limit social activities	N7: “I stopped going to my friends’ parties because I was afraid they would see me sick.”
	Impact on learning and work	N12: “I stopped going to school when I was diagnosed with cancer. “ N9: “I have not gone to work in a long time. My family does not allow me to do housework, much less go to work.”
	Impact on interpersonal relationships	N2: “I become very irritable, and always lose my temper. My husband is afraid of me. I wasn’t like this before.”

#### 3.1.1 Diversity of symptom types

The patient reported a number of physical symptoms that did not occur in isolation, but were intertwined to form complex symptom clusters. These symptom clusters place a significant burden on the patient’s psychological state, leading to ongoing psychological distress and anxiety. For example, some patients experienced a co-occurrence of nausea, fatigue and loss of appetite, which affected not only their physical health but also their mental health.

#### 3.1.2 Dynamics of symptom experiences

The dynamics of symptom experience are demonstrated by individual differences, inconsistency in symptom onset, and cyclical changes. The patient’s experience of symptoms is not static, but fluctuates with different stages of the chemotherapy cycle. This volatility requires continuous assessment of the patient’s symptoms and timely intervention by the healthcare team.

#### 3.1.3 Interference with daily life

Symptoms cause significant disruption to patients’ daily lives, affecting their ability to care for themselves, socialise, study and work, and their relationships. For example, some patients have had to reduce their social activities because of fatigue and nausea, or have become dependent on family members for care because of their reduced ability to do household chores.

### 3.2 Influencing factors of symptom experiences

This section summarises 6 first level themes and 20 s level themes to provide a more comprehensive understanding of the factors that influence patients’ experience of symptoms. Details are shown in Table 4.

**Table 4:** Influencing factors of symptom experiences.

First-level theme	Second-level theme	Patient quote
Individual differences	Younger patients experience more severe psychosocial effects	N12: “I’m only 17. Why did I get this disease? I do not want to die! I want to go to school.” (crying)
	Personality traits influence perception of and response to symptoms	N3: “I came in this time for a review. There is a little metastasis. I did not overthink it. Mindset is very important. Some people overthink, and the symptoms become more serious.”
	Cognitive level affects symptom judgement and coping	N1: “I sometimes get questions in my head. For example, I suddenly feel panicky and my heart races. I wonder, what is going on? I do not know what these symptoms mean; I have no concept of it.”
	Sensitivity to and tolerance of symptoms affect symptom experience	N7: “I find that even mild pain feels particularly intense to me. I need to be more aware of my body’s signals and find relief that works for me.”
Disease and treatment-related factors	Stage of treatment and disease progression Influence symptom experience	N4: “Since my cancer has spread, I’ve noticed more pain and discomfort. It’s harder to manage than before.”
	Overall health status influences complexity of symptom experience	N6: “I have other health issues besides cancer, and they definitely affect how I feel. It’s hard to separate the symptoms sometimes.”
	Patients feel acute symptoms more intensely	N2: “The last time I had CTX, I had diarrhea more than ten times, and I was about to lose consciousness. I told the doctor that I did not want to continue with the treatment.”	Different chemotherapy regimens affect incidence of symptoms	N2: “I had an allergy to Avvitine, which caused a rash and asthma. My doctor changed my treatment plan to irinotecan, but now I’m experiencing diarrhea again.”
	Cycle of chemotherapy affects severity of symptoms	N8: “I had no ill effects from my first two chemo treatments. I ate and slept well. The third time, I started vomiting. It wasn’t the same as before.”
	Temporal variations	Symptoms vary throughout the day	N4: “I find that the nausea and dizziness are worst when I wake up every morning, but they subside in the afternoon.”
Symptoms vary seasonally		N5: “The numbness and tingling in my hands and feet is worse in winter.”
Length of treatment affects symptoms		N7: “Since the 3rd CTX treatment, my hands and feet have become numb. But it was mild, so I just ignored it. Now after six times of CTX, it is becoming more and more painful.”
Daily habit	Sleep quality and symptoms interact	N9: “I find that if I do not sleep well at night, the side effects of CTX, such as nausea and tiredness, feel worse the next day.
	Eating right reduces chemotherapy symptoms	N6: “I’ve noticed that if I eat late or eat something fatty, the indigestion is more pronounced in the evening and the next morning.”
	Appropriate level of daily activity may relieve symptoms	N3: “After I started walking every day, I felt more energetic and less tired from chemotherapy.
Psychological and social support	Emotional management Helps reduce symptom distress	N11: “I started seeing a counselor after my diagnosis, and it’s made a world of difference. They’ve helped me cope with the emotional and psychological symptoms that were really affecting me.”	Family and social support positively impact symptom experience	N13: “If I feel my hands go numb, my wife massages them for me, so I do not feel much discomfort.”
	Healthcare team support and communication influence symptom interpretation and response	N10: “The nurse had told me my hands would go numb after using oxaliplatin. I was prepared for it.”
	Economic and social resources	Economic and insurance status affects symptom control	N8: “The hospital I was staying in was over 20,000 RMB per month out-of-pocket. I cannot afford it, so I just have to endure the discomfort.”
Access to and utilization of medical resources affect continuity of symptom management		N5: “The last time I tested positive for COVID-19, I had a high fever, cough, and could not walk. I missed my chemo appointment, stayed at home, and it turned into pneumonia.”

#### 3.2.1 Individual differences

Individual differences play an important role in the experience of symptoms. Younger patients may experience more severe psychosocial effects, such as anxiety about the future and a desire for education, as expressed by patient N12. In addition, individual personality traits, level of cognition, and sensitivity and tolerance to symptoms all influence how patients perceive and respond to symptoms.

#### 3.2.2 Disease and treatment-related factors

Disease stage and treatment progression have a significant impact on symptom experience. As the disease progresses, patients may experience more pain and discomfort, making symptoms more difficult to manage. Differences in chemotherapy regimens and changes in treatment cycles can also affect the onset and severity of symptoms.

#### 3.2.3 Temporal variations

The experience of symptoms varies from day to day and from season to season. For example, some patients report that nausea and dizziness are most severe when they wake up in the morning, while later in the day or during different seasons, certain symptoms may be less severe or more severe.

#### 3.2.4 Daily habits

Daily habits such as sleep quality, diet and level of daily activity interact with symptoms. A good night’s sleep and moderate physical activity can reduce feelings of fatigue, and a balanced diet can provide patients with sufficient nutrients. However, poor quality sleep or excessive physical activity can lead to physical fatigue if the patient does not sleep well, and an irregular diet can worsen indigestion.

#### 3.2.5 Psychological and social support

Emotional management, family and social support, and support and communication from the healthcare team all played a positive role in reducing symptom distress and improving symptom experience. For example, patient N11 significantly improved his emotional and psychological symptoms with the help of a counsellor.

#### 3.2.6 Economic and social resources

Access to and use of economic and social resources, such as health insurance status and access to healthcare resources, can also affect the continuity and effectiveness of symptom management. Financial burdens and resource constraints can lead to challenges in symptom management for patients.

## 4 Discussion

### 4.1 Focus on assessment and monitoring of CTX-related symptoms to improve symptom recognition

This qualitative study has revealed the multifaceted and dynamic nature of the symptoms experienced by patients with GI cancer undergoing CTX. Symptoms, which include nausea, anorexia, dyspepsia and diarrhoea, are not only influenced by tumour characteristics and the effect of CTX on the gut microbiota (Tumour and Microecology Professional Committee of the Chinese Anti-Cancer Association, 2022), but also significantly disrupt patients’ daily activities. In addition, diarrhoea is more common in people with gastrointestinal cancers, which may be related to the use of certain chemotherapy drugs (Sychev et al., 2021). These findings highlight the need for continuous monitoring of symptoms to facilitate timely intervention.

The study identified a spectrum of factors that patients perceived as influencing their symptom experience. We use the term ‘influencing factors’ with caution and emphasise that this does not imply a causal relationship, but rather reflects the subjective experience of patients during CTX. The epistemological limitations of qualitative research preclude the drawing of causal inferences from our data. Therefore, we advocate future research that integrates quantitative methods to more fully elucidate the manner in which these factors exert their influence on symptom experience.

Our analysis has revealed marked individual variability in the perception and response to symptoms, requiring a personalised approach to symptom assessment and management by the healthcare team. For example, psychological sequelae of CTX, such as anxiety and distress, are often more pronounced in younger patients due to concerns about their future and educational aspirations. In addition, variations in CTX regimens and progression through treatment cycles can lead to variations in the onset and severity of symptoms. Gender differences have been suggested as a factor in symptom experience, with discordance in the literature regarding symptom burden between male and female patients (Cheng and Lee, 2011; Pud, 2011; McCaughan et al., 2012). Notably, our study did not find significant differences in symptom presentation by gender, which would require individualised assessment and management of symptoms by the healthcare team.

The current paradigm of symptom assessment often relies on patient self-report of symptom severity to determine the need for clinical consultation (Maguire et al., 2021). This approach is prone to misinterpretation, potentially compromising patient safety (Molassiotis et al., 2019; Miller et al., 2020). Therefore, we propose a paradigm shift towards continuous, proactive assessment to ensure optimal symptom management. It is imperative that healthcare teams recognise the heterogeneity of patient symptoms and develop symptom-specific assessment protocols that are informed by an understanding of CTX and tailored to individual patient characteristics. Nurse-led initiatives such as admission symptom screening and pain assessment are integral to this process (Witzke et al., 2023). The dynamic assessment and intervention of symptom experience is most effectively delivered through a collaborative, multidisciplinary approach.

### 4.2 Improving doctor-patient communication and social support to optimise patient experience

This study highlights the central role of doctor-patient communication and social support in optimising the symptom experience of chemotherapy patients. Patients urgently need access to accurate and understandable medical information and crave emotional support from healthcare professionals and family members (Tsandila Kalakou et al., 2021). Existing studies have shown that increased social support is positively associated with significant improvements in patient symptoms (Khalid et al., 2023). In our study, the majority of cancer patients received significant support from family and friends, which was critical to their coping with the disease. However, there were also patients who expressed feelings of isolation and a greater need for professional support, including communication, practical help and advice from healthcare professionals. Currently, there are significant gaps in communication between healthcare professionals and cancer patients regarding disease information, treatment options, and symptom management (Kudjawu and Agyeman-Yeboah, 2021).

In light of this, we emphasise the need for healthcare professionals to improve communication with patients and provide comprehensive disease-related information, choice of treatment options and guidance on symptom management. In addition, daily living habits, including sleep quality, dietary habits and daily activity levels, are closely related to the severity of chemotherapy-induced symptoms. Therefore, we recommend that healthcare professionals provide guidance on healthy lifestyles and advise patients on how to reduce the impact of chemotherapy-related symptoms by modifying their daily living habits.

In addition, social support resources such as family, friends and peers should be actively mobilised to help patients develop individualised symptom management plans and provide ongoing health education. At the same time, improving the health insurance system, optimising the patient care process and helping patients to develop the positive beliefs needed to fight the disease in a holistic way are essential to improve their overall coping ability (Deng et al., 2022). Community health service providers should also strengthen the standardisation of chronic disease management, improve the quality of patient follow-up education, and conduct targeted group activities to actively disseminate symptom management information to further optimise patients’ symptom experience.

### 4.3 Exploring intelligent management methods to improve the efficiency of healthcare services

With the rapid development of health information technology, network hospitals and telemedicine solutions play a crucial role in public health crises, especially pandemics. They have a significant impact on alleviating health resource constraints and limitations of traditional health services (Anthony, 2023). Studies have already shown that case management through telehealth platforms significantly improves patient adherence and complication management, with positive and far-reaching effects on improving patient health (Windari et al., 2023). However, many healthcare organisations still face challenges in managing IT-based healthcare platforms, such as unregulated management and inefficient use of the platforms (Madandola et al., 2023).

Therefore, we propose a more comprehensive and efficient IT healthcare management system. This system should focus on promoting the standardisation of online healthcare services, improving the use of telemedicine platforms, and facilitating the exchange and sharing of medical knowledge. Through the implementation of this system, we will be able to better meet the individual needs of patients in terms of self-symptom management and case tracking, and provide them with personalised treatment plans. In addition, ensuring that medical information is updated in real time will enable healthcare professionals to use the latest research findings to guide treatment and, through an effective coordination and management mechanism, improve the efficiency of medical resource allocation and service quality.

To further improve the quality of symptom management, we recommend tools such as the Patient-Reported Outcome Measurement Information System (PROMIS) (Grauner et al., 2021) for real-time monitoring and remote management of symptoms. Future research should explore the use of mHealth technology and biomarkers to predict and assess symptom severity, leading to a more positive symptom experience for patients.

Although telehealth services offer great convenience to patients, there are potential limitations in providing emotional support and social interaction. To address this issue, we suggest enhancing the emotional and social support elements of telehealth services through appropriate management strategies. Examples include training healthcare workers in skills to provide empathy and emotional support in remote settings, and developing intelligent chatbots and virtual assistants that can provide immediate emotional feedback and support. These innovations are designed to help patients feel the emotional care and social connection they need while using telehealth services. We believe this is an important direction for future research to further explore to ensure patients receive comprehensive emotional and social support, alongside efficient healthcare services.

## 5 Limitations

This study used qualitative methodology to explore individual experiences in depth through semi-structured interviews, which limits the potential to infer causal relationships from the data. Given the exploratory nature of qualitative research, the findings are not intended to be generalised to a wider group, but should be validated by quantitative means and future research with a more diverse sample. In this study, interviews were conducted only in hospitals and only with cancer patients themselves, and the lack of representativeness of the sample due to differences in geography, humanities, etc., may affect the credibility of our findings. We have purposefully recruited patients with different demographics and medical characteristics, partly alleviating this bias concern. We need to follow up with interviews on symptom management strategies, as well as interviews on the experience of symptom management from the perspective of family members and healthcare professionals, to provide a more comprehensive reference for the formulation of symptom management support programs.

## 6 Conclusion

In this study, we conducted in-depth interviews with 13 patients with gastrointestinal cancer who were receiving chemotherapy. It was found that patients experienced a wide range of symptoms and that their symptom experience showed dynamic changes that significantly affected their daily lives. Many factors influence patients’ symptom experience, including individual differences, disease- and treatment-related factors, temporal changes, daily habits, psychological and social support, and economic and social resources. Therefore, nurses need to focus on assessing and monitoring chemotherapy-related symptoms, improving symptom recognition, and improving doctor-patient communication. In addition, nurses should enhance social support, explore smart management approaches, and improve the efficiency of health services to optimise the patient’s symptom experience.

## Data availability statement

The original contributions presented in the study are included in the article/supplementary material, further inquiries can be directed to the corresponding author.

## Ethics statement

The studies involving humans were approved by Medical Ethics Committee, Longhua Hospital, Shanghai University of Traditional Chinese Medicine, China. The studies were conducted in accordance with the local legislation and institutional requirements. The participants provided their written informed consent to participate in this study.

## Author contributions

XH: Data curation, Investigation, Writing – original draft, Writing – review & editing. HY: Project administration, Supervision, Writing – review & editing. YQ: Data curation, Writing – review & editing.
